# Long term outcome of adolescent and adult patients with pineal parenchymal tumors treated with fractionated radiotherapy between 1982 and 2003 -- a single institution's experience

**DOI:** 10.1186/1748-717X-5-122

**Published:** 2010-12-26

**Authors:** Eva Maria Stoiber, Benjamin Schaible, Klaus Herfarth, Daniela Schulz-Ertner, Peter E Huber, Jürgen Debus, Susanne Oertel

**Affiliations:** 1 Department of Radiation Oncology, University Hospital Heidelberg, INF 400, 69120 Heidelberg, Germany; 2 Department of Radiation Oncology, Markus Krankenhaus, Wilhelm Epstein-Strasse 4, 60431 Frankfurt am Main, Germany; 3 Department of Radiation Oncology, German Cancer Research Center, INF 280, 69120 Heidelberg, Germany

## Abstract

**Background:**

To evaluate the effectivity of fractionated radiotherapy in adolescent and adult patients with pineal parenchymal tumors (PPT).

**Methods:**

Between 1982 and 2003, 14 patients with PPTs were treated with fractionated radiotherapy. 4 patients had a pineocytoma (PC), one a PPT with intermediate differentiation (PPTID) and 9 patients a pineoblastoma (PB), 2 of which were recurrences. All patients underwent radiotherapy on the primary tumor site with a median total dose of 54 Gy. In 9 patients with primary PB treatment included whole brain irradiation (3 patients) or irradiation of the craniospinal axis (6 patients) with a median total dose of 35 Gy.

**Results:**

Median follow-up was 123 months in the PC patients and 109 months in the patients with primary PB. 7 patients were free from relapse at the end of follow-up. One PC patient died from spinal seeding. Among 5 PB patients treated with radiotherapy without chemotherapy, 3 developed local or spinal tumor recurrence. Both patients treated for PB recurrences died. The patient with PPTID is free of disease 7 years after radiotherapy.

**Conclusion:**

Local radiotherapy seems to be effective in patients with PC and some PPTIDs. Diagnosis and treatment of patients with more aggressive variants of PPTIDs as well as treatment of PB needs to be further improved, since local and spinal failure even despite craniospinal irradiation (CSI) is common. As PPT are very rare tumors, treatment within multi-institutional trials remains necessary.

## Background

The pineal gland is an endocrine gland which is responsible for the synthesis and secretion of melatonin. Tumors originating from the pineal region are very rare. They account for less than 1% of all primary central nervous system tumors [1]. They represent a very heterogeneous pathologic collective. Many types of central nervous system tumors like gliomas, meningeomas, choroid plexus papillomas and ependymomas can occur in the pineal region. The most common tumors in the pineal region, however, are germ cell tumors. This tumor entity constitutes about 21-70% of all pineal region tumors.

Pineal parenchymal tumors (PPT) are the second largest subgroup of pineal tumors and represent about 10-30%. They derive from pinealocytes, the cells the pineal gland is predominantly composed of. According to the WHO classification for tumors of the central nervous system revised in 2007, PPTs are subdivided into well-differentiated pineocytomas (PC), PPT with intermediate differentiation (PPTID) and poorly differentiated pineoblastomas (PB) [2]. In general, survival of patients with PPT is considered much more doubtful compared to that of patients with other pineal region tumors [1].

Among the PPT, PC are considered the slowest-growing with the most favorable overall survival rates. In contrast, PB represent the most primitive form of all PPT and histologically correspond to WHO grade IV tumors. They are one category of the heterogeneous, aggressive group of supratentorial primitive neuroectodermal tumors (sPNETs). Local recurrence and spread through the cerebro-spinal fluid tract are common. Due to the rare incidence of PPT there is ongoing debate regarding their therapeutic management. We retrospectively evaluated the long-term outcomes of our adolescent and adult patients with pineal parenchymal gland tumors treated with radiation therapy, to identify outcome, possible prognostic factors and patterns of failure in our patients.

## Materials and methods

### Record review

We performed a retrospective analysis including all patients with the histological diagnosis of a pineocytoma, PPT with intermediate differentiation or a pineoblastoma, who have been treated at our department of radiation oncology since 1982. Clinical data were collected, including age, extent of disease at diagnosis, initial treatment including resection status and details of radiation therapy (fields and doses) as well as information regarding recurrence pattern. In case of relapse, salvage treatment was documented. Information concerning early and late side effects were collected from the patient files. The extent of surgical tumor resection was assessed from the surgical and pathological reports and from postoperative imaging. If there was no evidence of contrast-enhancing tumor left on the postoperative imaging, patients were assigned a "Gross total resection (GTR)". "R2-resection" was defined as any surgical tumor resection less than GTR. The third category was "Biopsy only", for patients considered inoperable, when a biopsy only was performed for obtaining the tumor histology. For documentation of spinal seeding Chang M-stage classification was used. CSF cytology sampling was carried out inconsistently, especially in the 1980s and early 1990s, and was therefore not available in most patients. Time of disease progression was calculated from the end of radiotherapy treatment to the date of radiological local or cerebral/spinal tumor progression. Overall survival and local control rates were calculated from the end of radiotherapy treatment. Endpoint of the analysis was either the date of last follow-up or date of death.

### Patient demographics and previous treatment

Fourteen patients with PPT were treated between 1982 and 2003. 4 patients suffered from a pineocytoma, 1 from a PPTID and 9 patients from a pineoblastoma, 2 of which were recurrences. 10 patients were female, among them 2 with a pineocytoma and the patient with the PPTID. The median age at time of radiation treatment was 40 years (19-47 years) for the PC patients and 22 years (range 14-57 years) for the pineoblastoma patients (table 1). The patient with the PPTID was 25 years old at the time of treatment. At presentation, 3 of the patients with pineoblastoma had evidence of cerebral or spinal seeding (M+ disease). None of our patients suffered from extra-neural metastases. In all patients stereotactic biopsy for histological confirmation had been performed. None of the pineocytoma, but 4 of the patients with pineoblastoma had undergone gross or partial tumor resection. Two out of the 7 patients with primary pineoblastoma received chemotherapy prior to radiotherapy: A 17 female year-old patient (patient 11) was treated with chemotherapy according to the HIT 89 protocol after R2-resection. No cerebral or spinal seeding was present at time of diagnosis. One 14 year-old girl (patient 12) with spinal seeding at time of diagnosis received chemotherapy according to the HIT SKK 2000 protocol first, followed by autologous stem cell transplantation and percutanous radiation treatment afterwards.

**Table 1:** Patient characteristics

Patient	Age (Gender)	Histology	Year	**M**^**1)**^	Resection	Previous treatment	**RT field**^**2)**^	Total dose (boost) [Gy]	Alive (Recurrence)	Follow-up (Time to Progression) [months]
1	19 (m)	PC	1988	0	B	-	GTV	50	yes (no)	242
2	44 (f)	PC	1995	0	B	-	CTV	14 (56)	yes (no)	172
3	52 (f)	PC	1999	0	B	-	CTV	40 (54)	no (yes)	LF+SF(46)
4	37 (m)	PC	2003	0	B	-	GTV	54	yes (no)	71
5	25 (f)	PPTID	2002	0	B	-	GTV	54	yes (no)	84
6	40 (f)	PB	1990	0	R2	-	CSI	35 (50)	no (yes)	SF/LF(47/89)
7	17 (m)	PB	1982	0	B	-	WB	40 (60)	yes (no)	321
8	57 (m)	PB	1995	0	B	-	WB	34 (54)	yes (no)	3
9	28 (f)	PB	1996	0	R2	-	CSI	36 (52)	yes (yes)	LF (59)
10	22 (f)	PB	2000	0	R2	-	CSI	32 (59)	yes (yes)	LF+SF (13)
11	16 (f)	PB	1990	0	R2	CHT (HIT 89 Protocol)	WB	30 (52)	yes (no)	250
12	13 (f)	PB	2001	3	B	CHT (HIT 2000 Protocol)	CSI	40 (60)	no (yes)	SF (18)
13	14 (f)	PB	1995	2	B	iodine-seed BT	CSI	34 (54)	no (yes)	LF (12)
14	25 (f)	PB	1997	2	B	iodine-seed BT	CSI	36 (56)	no (yes)	LF (6)

^1) ^M stage prior to external beam radiation, ^2) ^star indicates additional boost to the primary tumor site, GTV: gross tumor volume, CTV: clinical tumor volume, WB: whole brain, CSI: craniospinal irradiation, B: Biopsy only, R2: any surgical resection less than gross total resection. LF: local failure, SF: Spinal failure

Two patients with pineoblastoma were treated for tumor recurrence. Both had previously undergone interstitial brachytherapy in other departments and were now treated with CSI plus local boost: A 14 year-old patient (patient 13) was treated for local tumor recurrence after initial treatment with iodineseed brachytherapy (TD 60 Gy) 7 months earlier. This patient showed additional cerebral tumor manifestation on MRI at the time of re-radiation. A second, 25 year-old woman (patient 14) was treated in our department after previous iodine-seed brachytherapy twice at two different tumor sites, 4 years and 10 months earlier. Both times, a TD of 60 Gy each had been administered, first to the pineal gland tumor and later to a suprasellar metastasis. Prior to both brachytherapy approaches a stereotactic biopsy had been performed, which revealed the histological diagnosis of a pineocytoma in both lesions. Prior to the percutaneous radiation treatment a third biopsy was taken, this time histology proved a pineoblastoma in the pineal lesion. In this patient, there was also evidence of cerebral seeding at the left temporal lobe at the time of fractionated radiation treatment.

### Radiotherapy

All patients were treated with percutaneous fractionated radiotherapy, none with radiosurgery. Single doses varied between 1,8 and 2 Gy (median 2 Gy) in all patients. For the pineocytoma patients the median administered radiation dose to the primary tumor site was 54 Gy (50--56 Gy). None of the patients received radiation to the whole brain or craniospinal axis in the primary setting.

In the patient with pineocytoma with intermediate differentiation, the gross tumor volume was irradiated up to a TD of 54 Gy in single fractions of 1.8 Gy.

Among the 7 patients with primary pineoblastoma, 3 received whole brain irradiation (patients 7, 8 and 11), in 4 patients the whole craniospinal tract was treated. Irradiation of the whole brain or the craniospinal tract was performed with a median dose of 35 Gy (30-40 Gy). The craniospinal axis was also treated in both patients with pineoblastoma recurrences. In all pineoblastoma patients a boost to the pineal tumor with a median TD of 54.2 Gy (50--60 Gy) was administered. Patient 12 additionally received a boost to the spinal metastasis (TD 50 Gy).

## Results

### Outcome in patients with pineocytoma and PPTID

The median duration of follow-up was 123 months (10.25 years) for the 4 pineocytoma patients (range 71--242 months). At the end of follow-up 3 patients were still alive and remained free of disease. Patient 3 developed late cerebral and spinal tumor seeding 46 months after radiation treatment and underwent salvage radiotherapy to the spinal axis with a TD of 39.6 Gy. She died 28 months later due to progressive disease.

The patient with pineocytoma with intermediate differentiation has remained free of disease for 84 months (7 years).

### Outcome in pineoblastoma patients

The median duration of follow-up was 109 months (9.1 years, range 3--321 months) for the 7 patients with primary pineoblastoma. Out of 5 patients who received radiation therapy alone, one patient developed a distant failure, one developed a local recurrence, and one patient developed both. All patients had received radiation treatment to the whole neuroaxis (<36 Gy). Both patients (6 and 9) who developed local failures had received a dose of less than 54 Gy to the local tumor. One patient (10) developed a distant failure despite treatment of the neuraxis with 32 Gy.

Among the 2 patients who were treated according to a multimodal treatment protocol, one patient (12) with M1 disease at presentation relapsed and died. The second patient (patient 11) is fine 250 months after treatment. Both patients (13 and 14) treated with CSI for pineoblastoma recurrences after initial iodine-seed brachytherapy died.

Patient 6 presented with a spinal metastasis 47 months after radiation treatment and also developed local recurrence in the pineal region 89 months after RT. This patient was initially treated with a TD of 35 Gy to the neuraxis. Salvage treatment of the local tumor recurrence consisted of iodine-seed brachytherapy, the spinal metastasis in the lumbar region was re-radiated percutaneously (TD 19,8 Gy). 18 months later the patient received chemotherapy (cisplatin and etoposid) due to progressive disease, which was switched to gemcitabine 14 months later. The patient died shortly afterwards due to further tumor progression.

Patient 7 received radiotherapy only and has remained free of disease.

Patient 8 was lost to follow-up 3 months after radiation treatment.

Patient 9 developed local tumor recurrence in the pineal region 59 months after initial treatment of the neuraxis. The patient was re-irradiated locally (TD 36 Gy) and is alive and free of disease 123 months after radiation treatment.

Patient 10 developed diffuse cerebral and spinal tumor recurrences 13 months after treatment. Salvage chemotherapy was successful and the patient is still alive 31 months later.

Patient 11 has remained free of disease 250 months after multimodal treatment.

Patient 12 suffered from cerebral and spinal tumor recurrence 18 months after initial multimodal treatment. In the following 14 months she received different salvage chemotherapy regimens with Mafosfamid first, followed by temozolomide and Roaccutan. Despite reirradiation to a metastasis at the cavernous sinus (TD 57,2 Gy) and in the lumbar spine (TD 30 Gy) the patient died 6 months after completion of RT from tumor progression.

Patient 13 developed cerebral tumor progression 12 months after treatment with fractionated radiotherapy. Salvage chemotherapy (PCV) was initiated, but the girl deceased 6 months later.

Patient 14 developed tumor progression of the suprasellar tumor 6 months after fractionated radiation therapy. Reirradiation with stereotactic radiosurgery was performed (TD 12.5 Gy@80% isodose). The patient also developed spinal tumor seeding 11 months later and died shortly afterwards.

### Toxicity

There were no severe early or late side effects due to fractionated local radiation therapy observed in the patients with pineocytoma. In two patients visual impairment improved substantially until the first follow-up visit 3 months after treatment. 2 of the 9 pineoblastoma patients suffered from sepsis due to leucopenia during CSI. No neurotoxicity was observed. Both patients <14 years treated with CSI died within 2 years after treatment, therefore no patient was at risk for growth retardation in our collective. One Patient (7) had to be treated for complete panhypopituitarism 40 months after whole brain irradiation with boost.

## Discussion

While fractionated local radiotreatment of pineocytomas seems to be a safe and effective method with a local control rate of 100% in our small collective, intensification of therapy in aggressive variants of intermediate pineocytomas as well as pineoblastoma seems necessary. Despite the small patient cohort, the results of this analysis support those of other published series.

### Impact of exact histological grading

In terms of overall survival and disease relapse 3 of 4 patients primarily diagnosed with pineocytoma could be cured by local radiotherapy only, whereas curation by radiation only failed in most patients with pineoblastomas, even in case of CSI. Considering that also one of our patients treated for pineocytoma experienced spinal seeding, therefore suggesting a more aggressive tumor variant, and that two of the patients referred with a pineoblastoma had primarily been diagnosed with pineocytoma years earlier, the difficulty and necessity of exact histological grading prior to the choice of treatment, including the size of radiation fields becomes once more apparent. Careful histopathological classification is required in order to identify mixed- or intermediate typed pineocytomas. The exact differentiation of these intermediate typed pineocytomas, formerly also referred to as mixed pineocytoma-pineoblastoma, however, remains difficult [3,4]. After correlation of histopathological findings and prognosis of 66 patients with PPT Jouvet et al [3] have suggested a further differentiation amongst the PPTIDs, differing those with less than 6 mitoses and positive immunolabeling for neurofilaments (less aggressive) from those with 6 or more mitoses and a lack of neurofilament staining (more aggressive).

In the pineocytoma patients of our series histological diagnosis was reached by stereotactic biopsy only, none of the patients underwent open resection. This increases the likelihood of misdiagnosis, since in small tissue samples potentially less-differentiated tumor cells might be missed. This may also explain the disparity between the suspected benign histology and the aggressive effects in the patients mentioned above. Obtaining a maximal amount of viable tissue for pathological examination is crucial for accurate histological diagnosis of a pineocytoma.

### Impact of extent of disease

The impact of extent of disease in posterior fossa PNETs (e.g. medulloblastoma) is well known, but it is less well studied for sPNETs like PB. However, Fauchon et al [5] previously confirmed, that also in PPTs the local tumor volume is a significant prognostic factor. Tumor size influenzed both survival and the risk of perioperative complications in Fauchon's study. Radical surgery did not improve survival significantly, however, a trend towards a longer disease-free survival after complete resection was seen. For children with PB, an older study of the children's cancer group (CCG-921) showed a clear negative impact of M1+ at presentation [6]. Nevertheless few authors noted that favourable outcomes for a subset of M+ patients treated are possible [7,8]. In our analysis, the patients initially presented with M+ disease showed impaired overall survival.

### Recurrent Pineoblastomas

Both patients treated for tumor recurrence after initial treatment with interstitial brachytherapy died, even though the administered radiation dose was similar to the dose prescribed in the primary setting. Some newer treatment protocols for recurrent sPNET use surgery and chemotherapy to minimize residual disease first, followed by high dose chemotherapy and autologous stem cell rescue as a possible curative salvage treatment [9]. However, so far reported survival outcome for patients with recurrent PB has remained poor, even though curation seems to be possible (patient 9 and 10). It should be noted here that previous treatment with interstitial brachytherapy, however, did not eliminate the possibility of later external beam irradiation in these cases of tumor recurrence.

Due to the rare incidence of PPT, most of the published series analyzed long time periods retrospectively. Therefore differences in workup, diagnosis and treatment modalities are unavoidable and comparison of the data of a long time period is difficult. PPT are not a homogeneous histological tumor entity. According to the 2007 WHO Classification of tumors of the central nervous system they are classified into 3 groups: Pineocytomas, PPT with intermediate differentiation and pineoblastomas [2]. Many older published series separated PPT even in only two histological subtypes, PC and PB. No intermediate differentiated PPTs, classified as WHO grade II-III tumors, according to the new WHO classification system, were documented. Since there is a large pathologic variability among PPT it is difficult to draw general conclusions about their behavior. Local control, the potential to seed among the cerebro-spinal fluid path and overall survival varies widely among the heterogeneous collective of PPTIDs.

Among the PPT, pineocytomas are considered the slowest-growing with the most favorable overall 5- year survival rates. Histologically they represent WHO grade I tumors [2]. In patients treated with surgery and/or radiosurgery, 5-year overall survival rates of up to 90% have been reported [7,8]. Publications on the use of sole local fractionated radiotherapy in the management of pineocytomas are rare. Several retrospective studies analyzing the long-term outcome of patients treated with radiosurgery exist. These studies give evidence that radiosurgery constitutes a comparably safe and very effective treatment for PPT [10-13] both as a tool in a multidisciplinary treatment approach, or as a single mean in selected cases. Tumor size decreases by up to 70% and even complete regressions have been described [14] as well as excellent local tumor control rates of up to 100% for small tumors [10]. In an older analysis with only five patients, Vaquero et al. [15] showed that postoperative radiation after surgical removal can be omitted, even in case of local invasion in PPTs with neuronal differentiation. Especially in older studies, pineocytomas, however, did sometimes not behave as benign as expected, particularly in younger patients [16-18]. Also among our four patients with pineocytoma one patient developed spinal seeding, thus suggesting a more aggressive tumor variant. Obtaining a maximal amount of tissue sample is therefore crucial in suspected pineocytomas, to decrease the likelihood of a histological misdiagnosis as a possible cause for this aggressive behavior. Kurisaka et al [16] were able to show, that additional radiotreatment does improve 5-year local survival in PPTIs from 83% to 43%, possibly because in the data held as PPTs by the All Japan Brain Tumor Registry aggressive PPTIDS may have been included. Yet, close follow-up including MRI of the neuraxis is recommended for all patients with PPT to detect recurrence at an early state, since successful salvage therapy is possible.

Pineoblastomas are classified as PNETs. The main indicators of prognosis for PB seem to be histopathological diagnosis and staging of the neuraxis [19]. Like other PNETs they tend to metastasize widely throughout the CSF pathway. Even the occurence of extra-neural metastases in PB patients has been described [20]. Since PNETs often relapse locally, it is also necessary to establish exact criteria for local RT planning [21]. In our cohort, 2 patients with primary pineoblastoma and both patients with pineoblastoma recurrences developed local failure. Up to date there is no generally accepted therapeutic approach in the primary treatment of pineoblastomas. Dose escalation within the pineal tumor (e.g. via a particle therapy boost or precision photon RT or radiosurgery) is currently one of the treatment strategies investigated in clinical trials. Excellent local control rates in patients who recieved doses equivalent to >50 Gy fractionated radiation, compared to patients treated with lesser doses have been observed by Schild et al [22]. But his study not only included PB patients, but also patients with PPT with intermediate differentiation, mixed tumors and pineocytoma.

Patients ≤21 years of age are usually included in the HIT-2000 protocol, which stratifies radiation therapy according to risk profile. E.g. in M+ patients, intensified sandwich chemotherapy is combined with hyperfractionated radiotherapy with local doses of up to 40 Gy to whole brain and craniospinal axis, and boosts up to 72 Gy locally. Published data also suggest that combined treatment consisting of gross tumor resection, CSI and multi-agent chemotherapy correlate with improved survival in pediatric pineoblastoma patients [7]. In order to reduce radiation induced late effects, other groups are investigating risk-adapted craniospinal radiotherapy as part of a multimodal treatment approach. E.g. Chintagumpala undertook a study to estimate the event free survival (EFS) of patients with newly diagnosed supratentorial PNETs treated with risk-adapted CSI with additional radiation to the primary tumor site and subsequent high-dose chemotherapy supported by stem cell rescue [23]. 7 of 16 enrolled patients had pineal PNETs, 5 of them with high-risk disease defined as metastatic disease in the brain or spine, or presence of residual disease >1.5 cm^2^. Patients received risk-adapted CSI with 23.4 Gy in case of M0 patients ("average-risk patients") and 36-39.6 Gy for highrisk patients. Overall, the 5-year EFS for all 7 pineoblastoma patients was 54% with an overall survival of 67%. The authors conclude that the doses to the craniospinal axis might be reduced in a subset of patients without compromising event-free survival [23].

## Conclusion

Despite its limitation by small patient numbers, the presented study adds further evidence to the importance of an improvement of histological diagnosis and classification. While local radiotherapy of patients with pineocytoma seems to be safe and effective, intensification of treatment in some PPTID and pineoblastoma patients by addition of chemotherapy and/or radiation therapy within clinical trials seems to be warranted, since local and spinal failures are common after RT. Close follow-up after treatment is important, since salvage therapies can be effective.

## Competing interests

The authors declare that they have no competing interests.

## Authors' contributions

EMS is responsible for data acquisition, literature research and writing of the manuscript. BS is responsible for data acquisition. PH, JD, KH and DSE are responsible for the clinical treatment of the patients and concepts. SO is responsible for literature research and proof reading. All authors read and approved the final manuscript.

## References

[B1] Al-Hussaini M Sultan I Abuirmileh N Jaradat I Qaddoumi I Pineal gland tumors: experience from the SEER database J Neurooncol 2009 94 351 358 10.1007/s11060-009-9881-9 19373436 PMC2804886

[B2] Louis DN Ohgaki H Wiestler OD Cavenee WK Burger PC Jouvet A Scheithauer BW Kleihues P The 2007 WHO classification of tumors of the central nervous systems Acta Neuropathol 2007 114 97 109 10.1007/s00401-007-0243-4 17618441 PMC1929165

[B3] Jouvet A Saint-Pierre G Fauchon F Privat K Bouffet E Ruchoux MM Chauveinc L Fèvre-Montange M Pineal parenchymal tumors: a correlation of histological features with prognosis in 66 cases Brain Pathol 2000 10 1 49 60 Review 10.1111/j.1750-3639.2000.tb00242.x 10668895 PMC8098427

[B4] Anan M Ishii K Nakamura T Yamashita M Katayama S Sainoo M Nagatomi H Kobayashi H Postoperative adjuvant treatment for pineal parenchymal tumour of intermediate differentiation J Clin Neurosci 2006 13 9 965 8 Epub 2006 Aug 14 10.1016/j.jocn.2005.11.036 16904896

[B5] Fauchon F Jouvet A Paquis P Saint-Pierre G Mottolese C Ben Hassel M Chauveinc L Sichez JP Philippon J Schlienger M Bouffet E Parenchymal pineal tumors: a clinicopathological study of 76 cases Int J Radiat Oncol Biol Phys 2000 46 4 959 68 10.1016/S0360-3016(99)00389-2 10705018

[B6] Jakacki RI Zeltzer PM Boyett JM Albright AL Allen JC Geyer JR Rorke LB Stanley P Stevens KR Wisoff J Survival and prognostic factors following radiation and/or chemotherapy for primitive neuroectodermal tumors of the pineal region in infant and children: a report of the children's cancer group J Clin Oncol 1995 13 6 1377 1383 7751882 10.1200/JCO.1995.13.6.1377

[B7] Gilheeney SW Saad A Chi S Turner C Ullrich NJ Goumnerova L Scott RM Marcus K Lehman L De Girolami U Kieran MW Outcome of pediatric pineoblastoma after surgery, radiation and chemotherapy J Neurooncol 2008 89 1 89 95 10.1007/s11060-008-9589-2 18415046

[B8] Gururangan S McLaughlin C Quinn J Rich J Reardon D Halperin EC Herndon J Fuchs H George T Provenzale J Watral M McLendon RE Friedman A Friedman HS Kurtzberg J Vredenbergh J Martin PL High-dose chemotherapy with autologous stemcell rescue in children and adults with newly diagnosed pineoblastomas J Clin Oncol 2003 21 11 2187 2191 10.1200/JCO.2003.10.096 12775745

[B9] Broniscer A Nicolaides TP Dunkel IJ Gardner SL Johnson J Jr Allen JC Sposto R Finlay JL High dose chemotherapy with autologous stem-cell rescue in the treatment of patients with recurrent non-cerebellar primitive neuroectodermal tumors Pediatr Blood Cancer 2004 42 3 261 7 10.1002/pbc.10369 14752864

[B10] Hasegawa T Kondziolka D Hadjipanayis CG Flickinger JC Lunsford LD The role of radiosurgery for the treatment of pineal parenchymal tumors Neurosurgery 2002 51 880 889 10.1097/00006123-200210000-00006 12234394

[B11] Mori Y Kobayashi T Hasegawa T Yoshida K Kida Y Stereotactic radiosurgery for pineal and related tumors Prog Neurol Surg 2009 23 106 18 full_text 19329865 10.1159/000210057

[B12] Kano H Niranjan A Kandziolka D Flickinger JC Lunsford D Role of stereotactic radiosurgery in the management of pineal parenchymal tumors Prog Neurol Surg 2009 23 44 58 full_text 19329860 10.1159/000210052

[B13] Manera L Regis J Chinot O Porcheron D Levrier O Farnarier P Peragut JC Pineal region tumors: The role of stereotactic radiosurgery Stereotact Funct Neurosurg 1996 66 164 173 10.1159/000099807 9032858

[B14] Reyns N Hayashi M Chinot O Manera L Peragut J-C Blond S Regis J The role of Gamma Knofe radiosurgery in the treatment of pineal parenchymal tumors Acta Neurochir 2006 148 5 11 10.1007/s00701-005-0626-z 16172830

[B15] Vaquero J Ramiro J Martínez R Coca S Bravo G Clinicopathological experience with pineocytomas: report of five surgically treated cases Neurosurgery 1990 27 4 612 8 discussion 618-9 10.1097/00006123-199010000-00018 2234367

[B16] Kurisaka M Arisawa M Mori T Sakamoto T Seike M Mori K Okada T Wakiguchi H Kurashige T Combination chemotherapy (cisplatin, vinblastin) and low-dose irradiation in the treatment of pineal parenchymal cell tumors Childs Nerv Syst 1998 14 10 564 9 10.1007/s003810050273 9840380

[B17] Marsh WR Laws ER Jr Shunting and irradiation of pineal tumors Clin Neurosurg 1985 32 384 396 3905146

[B18] Disclafani A Hudgins RJ Edwards MS Wara W Wilson CB Levin VA Pineocytomas Cancer 1989 63 302 394 10.1002/1097-0142(19890115)63:2<302::AID-CNCR2820630216>3.0.CO;2-D 2910435

[B19] Lutterbach J Fauchon F Schild S Chang S Pagenstecher A Volk B Ostertag C Momm F Jouvet A Malignant pineal parenchymal tumors in adult patients: patterns of care and prognosic factors Neurosurgery 2002 51 44 56 10.1097/00006123-200207000-00006 12182434

[B20] Frasen G Rampling R Smith C Nicoll J Stephan M Long term survival following extra-neural metastasis from a pineaoblastoma J Neurooncol 2000 48 141 144 10.1023/A:1006430022068 11083078

[B21] Taylor RE Donachie PH Weston CL Robinson KJ Lucraft H Saran F Ellison DW Ironside J Walker DA Pizer BL Impact of radiotherapy parameters on outcome for patients with supratentorial primitive neuro-ectomdermal umors entered into the SIOP/UKCCSG PNET 3 study Radiother Oncol 2009 92 83 88 10.1016/j.radonc.2009.02.017 19328574

[B22] Schild SE Scheithauer BW Schomberg PJ Hook CC Kelly PJ Frick L Robinow JS Buskirk SJ Pineal parenchymal tumors. Clinical, pathologic, and therapeutic aspects Cancer 1993 72 3 870 80 Review 10.1002/1097-0142(19930801)72:3<870::AID-CNCR2820720336>3.0.CO;2-X 8334641

[B23] Chintagumpala M Hassall T Palmer S Ashley D Wallace D Kasow K Merchant TE Krasin MJ Dauser R Boop F Krance R Woo S Cheuk R Lau C Gilbertson R Gajjar A A pilot study of riskadapted radiotherapy and chemotherapy in patients with supratentorial PNET Neuro Oncol 2009 11 1 33 40 10.1215/15228517-2008-079 18796696 PMC2718957

